# Core Muscle Activity during Physical Fitness Exercises: A Systematic Review

**DOI:** 10.3390/ijerph17124306

**Published:** 2020-06-16

**Authors:** José M. Oliva-Lozano, José M. Muyor

**Affiliations:** 1 Health Research Centre, University of Almería, 04120 Almería, Spain; jol908@ual.es; 2 Laboratory of Kinesiology, Biomechanics and Ergonomics (KIBIOMER Lab.), Research Central Services, University of Almería, 04120 Almería, Spain

**Keywords:** EMG, muscle activation, abdominal muscles, resistance exercises, strength, fitness

## Abstract

The aim of this study was to systematically review the current literature on the electromyographic (EMG) activity of six core muscles (the rectus abdominis, the internal and external oblique, the transversus abdominis, the lumbar multifidus, and the erector spinae) during core physical fitness exercises in healthy adults. A systematic review of the literature was conducted on the Cochrane, EBSCO, PubMed, Scopus, and Web of Science electronic databases for studies from January 2012 to March 2020. The Preferred Reporting Items for Systematic Reviews and Meta-analyses (PRISMA) guidelines were used. The inclusion criteria were as follows: (a) the full text available in English; (b) a cross-sectional or longitudinal (experimental or cohorts) study design; (c) the reporting of electromyographic activity as a percentage of maximum voluntary contraction (% MVIC), millivolts or microvolts; (d) an analysis of the rectus abdominis (RA), transversus abdominis (TA), lumbar multifidus (MUL), erector spinae (ES), and the internal (IO) or external oblique (EO); (e) an analysis of physical fitness exercises for core training; and (f) healthy adult participants. The main findings indicate that the greatest activity of the RA, EO, and ES muscles was found in free-weight exercises. The greatest IO activity was observed in core stability exercises, while traditional exercises showed the greatest MUL activation. However, a lack of research regarding TA activation during core physical fitness exercises was revealed, in addition to a lack of consistency between the studies when applying methods to measure EMG activity.

## 1. Introduction

Fitness is defined as a state of health and well-being, which is characterized by the ability to perform daily physical activities or exercise [1]. Thus, the primary purpose of strength and conditioning coaches is to prescribe the right physical fitness exercises to their athletes and/or clients in order to achieve specific fitness goals [2]. Several studies have provided information on the importance of core training and testing in several populations [3,4] in order to improve performance [5] and reduce the risk of injury (e.g., back and lower extremity injury) [6,7]. In addition, core physical fitness exercises may contribute to decreasing the risk of other musculoskeletal disorders (e.g., excessive load on lumbar spine, imbalance of hip extensors, atrophy of paraspinal muscles), which are the consequence of faulty postures and sedentary lifestyles [8].

The core is defined as an anatomical box which consists of several muscle groups, such as the rectus abdominis at the front, the internal and external obliques on the lateral sides, the erector spinae, lumbar multifidus, and quadratus lumborum at the back, the diaphragm at the upper edge and the pelvic floor, and the iliac psoas at the bottom [9,10]. From a practical perspective, the core muscles are the center of the body where most kinetic chains transfer forces to the extremities [10]. However, the transversus abdominis, lumbar multifidus, and quadratus lumborum are considered the key core muscles for fitness and health professionals [2]. 

In recent years, the development of surface electromyography (sEMG) has allowed us to measure muscle activation patterns [11]. These muscle activation patterns should be considered when selecting and prescribing physical fitness exercises [12], since the force of the muscle contraction is regulated by the totality of motor units recruited [13,14]. In addition, the recruitment of low- or high-threshold motor units depends on the intensity of the exercise [14]. Thus, the amplitude of the sEMG signal, which is frequently reported as raw (millivolts) or relative to the maximum voluntary isometric contraction (% MVIC), is commonly used to analyze levels of muscle activation and fatigue [2,15]. Given that the greater the electromyographic (EMG) activity, the greater the challenge to the neuromuscular system, it is suggested that the core exercises that increase EMG may be useful for core strengthening [2].

Sit-ups and curl-ups have, for a long time, been the most common core physical fitness exercises [16]. However, new exercises have been developed by adding, for instance, unstable surfaces, such as Swiss balls, BOSU balls, or wobble balance board platforms in order to increase the proprioceptive demands of the exercises [16]. In addition, a recent systematic review on the EMG activity during core physical fitness exercises considered that free-weight exercises may be recommended, since these multi-joint exercises are more time-efficient than core exercises on the floor or on unstable surfaces [2]. Nevertheless, research to date has been limited on which core exercises should be performed based on muscle activity patterns, and there is a discernible lack of consensus. The only review on core muscle activity in physical fitness exercises for healthy adults was published seven years ago, in which the authors concluded that fitness specialists should focus on free weight exercises (e.g., the squat or deadlift) rather than other specific core exercises in order to train these muscles [2]. However, this review only included studies that analyzed the muscle activity of three core muscles (the transversus abdominis, lumbar multifidus, and quadratus lumborum) [2]. In addition, new exercises have since been evaluated (e.g., the suspension plank, roll-out, body saw, pike, and knee tuck) [17,18,19] so the literature is in need of an updated systematic review. 

Consequently, the aim of this study was to systematically review the current literature on the electromyographic activity in six core muscles (the rectus abdominis, internal and external oblique, transversus abdominis, lumbar multifidus, and erector spinae) during core physical fitness exercises in healthy adults.

## 2. Materials and Methods

### 2.1. Search Strategy

A systematic review of the literature was conducted on the Cochrane, EBSCO, PubMed, Scopus, and Web of Science electronic databases, looking at studies from 12 January 2012, when the last systematic review was performed [2] up until 5 March 2020. The Preferred Reporting Items for Systematic Reviews and Meta-analyses (PRISMA) guidelines [20] were used. The protocol for this systematic review was registered on PROSPERO (CRD42020176876) and is available in full on the National Institute for Health Research. 

The keywords for the search strategy were (“core” OR “trunk” OR “abdominis” OR “abdominal” OR “low back” OR “rectus abdominis” OR “transversus abdominis” OR “multifid*” OR “lumbar” OR “quadratus lumborum” OR “erector spinae” OR “external oblique” OR “internal oblique”) AND (“resistance training” OR “strength training” OR “resistance exercise” OR “weight lifting” OR “weight-bearing” OR “stability” OR “strengthening” OR “training”) AND (“electromyography” OR “EMG” OR “muscle activation” OR “biofeedback” OR “myoelectrical”).

### 2.2. Study Selection

Only studies meeting the inclusion criteria were selected. These criteria were as follows: (a) the full text being available in English; (b) a cross-sectional or longitudinal (experimental or cohorts) study design; (c) a report on the electromyographic activity as a percentage of maximum voluntary contraction (% MVIC), millivolts or microvolts; (d) an analysis of the rectus abdominis (RA), transversus abdominis (TA), lumbar multifidus (MUL), erector spinae (ES), and internal (IO) or external oblique (EO); (e) an analysis of the physical fitness exercises for core training; (f) inclusion of healthy adult participants; and (g) being published as of 1st March 2020. Any studies that included a history of low back pain, spinal injury, or neurological deficits in any of the participants were excluded, along with any that included an analysis of aerobic exercises, books, theses, and/or congress abstracts.

**Figure 1:**
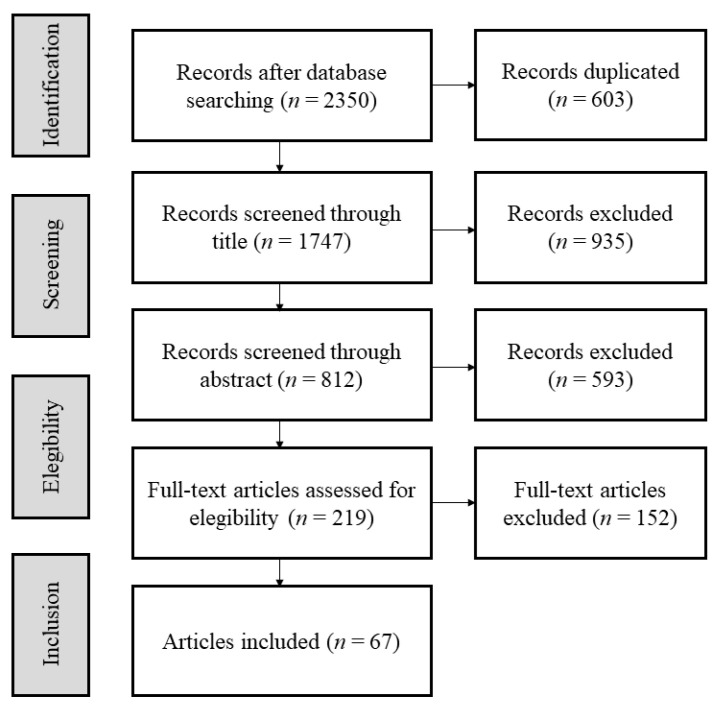
Flowchart of the selection process.

The studies were selected by two independent reviewers based on the inclusion and exclusion criteria. All of these were stored in the Mendeley reference management system (Elsevier, Amsterdam, The Netherlands). Once the duplicates were removed, the titles and abstracts were examined. Following this, the full text of all the papers were read, and only studies meeting the inclusion criteria were selected. In the case of any disagreement between the two reviewers, a third collaborator participated in the decision-making process. Figure 1 shows a graphical description of the study selection process, which lasted for three weeks.

### 2.3. Data Abstraction

The following data were extracted from each study: the authors, country, year, sample size, gender, age, exercise(s) assessed and methods used, the muscles tested, the results (in % MVIC, microvolts or millivolts for each muscle during the exercise), and the conclusion. If a study reported the results in microvolts, it was converted into millivolts. Given the degree of heterogeneity between the studies (e.g., the sample characteristics, data collection methods, electrodes placement, data reporting in different units of measure), the data collected in this systematic review could not be used for the purposes of meta-analysis. For this reason, a systematic qualitative review and interpretation of the results was carried out.

### 2.4. Core Physical Fitness Exercises

The core exercises were based on prior classifications [2], these being: (a) traditional core exercises—low-load exercises which are usually performed on the floor in order to activate superficial muscles (e.g., the sit-up and back extension); (b) stability exercises—low load and low range of motion in order to activate deep core muscles (e.g., the front plank and side plank); (c) ball/device exercises—a combination of stability and traditional core exercises which might add unstable surfaces or devices (e.g., a crunch on a Swiss ball and the front plank on suspension systems); and (d) free-weight exercises—the addition of greater loads which tend to activate the upper or lower body and core muscles (e.g., the squat, deadlift, and shoulder press). 

### 2.5. Methodological Quality Assessment

The Effective Public Health Practice Project (EPHPP) scale was used to assess the level of evidence of each study. Currently, there is no standard scale for the methodological quality assessment of observational investigations on EMG [21,22]. However, each study was assessed based on the EPHPP scale, which has been used as a standard tool for the assessment of methodological quality in previous research with similar aims [2,21]. In addition, the use of this scale may decrease the risk of bias when interpreting the results from this systematic review. The EPHPP scale has six components (selection bias, study design, confounders, blinding, data collection method, and withdrawals/dropouts) categorized by three ratings (weak, moderate, and strong) [23]. The level of evidence of each paper may be weak (two or more weak ratings), moderate (one weak rating), or strong (no weak ratings). Once the studies were included in this systematic review, two reviewers rated each study. If there was any hesitation or question related to one of the components being rated, the reviewers discussed: oversight (final decision: strong), different criteria interpretations (final decision: moderate), and different study interpretations (final decision: weak).

## 3. Results

### 3.1. Study Selection

A total of 2350 studies were identified following the search strategy; however, 603 were duplicates. Once these were removed, titles and abstracts were examined, and 219 were selected for full-text screening. Of these, 152 did not meet the inclusion criteria (e.g., most of these studies did not meet healthy adult participant criteria) so the remaining 67 studies were selected for the study (Figure 1).

### 3.2. Characteristics of the Selected Studies

A total of 1247 healthy participants were analyzed in all the selected studies (Table 1). There were studies that collected data from male-specific samples (*n* = 40), female-specific samples (*n* = 9), and samples from both genders (*n* = 18). The EMG activity from 233 exercises was collected. Table 2 shows a summarized description of the exercises. Based on prior core exercise classifications [2], 15 studies analyzed traditional core exercises, 23 analyzed core stability exercises, 26 analyzed core exercises with an additional ball/device, and 26 analyzed free-weight exercises (Table 1). Each study measured the EMG activity in different muscles: the RA (*n* = 51), EO (*n* = 45), ES (*n* = 37), IO (*n* = 23), MUL (*n* = 16), and TA (*n* = 3). 

The methods used to measure EMG activity varied depending on the study, although the most frequent methods were: three sets of 5 s (*n* = 9), five repetitions (*n* = 6), three sets of 10 s (*n* = 5), 10 repetitions (*n* = 5), and six maximum repetitions (*n* = 4). This systematic review included studies with strong (*n* = 20), moderate (*n* = 38), and weak (*n* = 9) levels of evidence. In addition, a lack of unified criteria was found when reporting EMG values, since studies used % MVIC (*n* = 55) (Table 3), millivolts (*n* = 8), and microvolts (*n* = 4) (Table 4). 

### 3.3. Rectus Abdominis

Regarding the traditional core exercises, the static curl-up with the hands behind the neck, the hip flexed at 60°, and knees flexed at 90° was the exercise that elicited the highest EMG activity of the RA (81.00 ± 10.90% MVIC), followed by the static curl-up with the arms crossed over the chest, the hip flexed at 60°, and knees flexed at 90° (67.60 ± 15.70% MVIC) [24]. Based on the EMG activity reported as mV, the exercise with the highest RA activation was the sit-up, with lower RA activation being higher than upper RA activation (0.54 ± 0.24 mV vs. 0.27 ± 0.11 mV) [16].

V-sits [25], the front plank with scapular adduction and posterior pelvic tilt [26], and the side plank with maximum expiration [27] were the core stability exercises with the greatest % MVIC in the RA (V-sits: ~80%; Front plank: ~78%; Side plank: ~75%). The front plank with additional weight (20% BW) also showed the greatest mV (~0.25 mV) in the RA [28].

Three studies reported the following core exercises on a ball/device as the highest EMG related to % MVIC in the RA: the suspended roll-out plank (Upper RA: 145.00 ± 22.00% and Lower RA: 122.00 ± 32.00%; Upper RA: 67.00 ± 78.00% and Lower RA: 140.00 ± 89.00%) [19,29] and the suspended front plank (Upper RA: 145.00 ± 22.00% and Lower RA: 122.00 ± 32.00%) [29]. For those studies that reported EMG activity as mV, the highest values were obtained for sit-ups with upper instability on a BOSU ball (Upper RA: 0.33 ± 0.14 mV; Lower RA: 0.65 ± 0.33 mV) [16]. 

The unstable Bulgarian squat and the regular back squat over six maximum repetitions were the free-weight exercises with the highest EMG activity (~210% MVIC) [30].

### 3.4. Internal Oblique

Regarding traditional exercises, the static curl-up with the hands behind the neck, the hip flexed at 60°, and the knees flexed at 90° and at 45° was the exercise with the highest % MVIC (without twist: 61.70 ± 17.00% MVIC; with twist: 57.30 ± 12.40% MVIC) [24]. The crunch was the exercise with the highest mV values (~0.08 mV) [31].

In relation to core stability exercises, the front plank with scapular adduction and posterior pelvic tilt had the highest % MVIC for IO (119.92 ± 60.26% MVIC) [26], while the climax laughter exercises showed the highest mV values (~0.11 mV) [31].

The core exercises with the greatest activity performed on an additional ball/device were the front plank on a Swiss ball with hip extension (76.50 ± 37.00% MVIC) and the stir-the-pot (73.50 ± 31.30% MVIC) [32]. There were only three studies that examined IO activity during free-weight exercises [33,34,35], with the highest EMG values obtained on the kettlebell swing with kime (80.80 ± 43.70% MVIC) and the unilateral bench press (~0.05 mV).

### 3.5. External Oblique

The curl-up with the hip flexed at 90° had the highest activation within the traditional core group (with maximal expiration: 70.74 ± 20.57 % MVIC; with slow expiration: 65.18 ± 24.83 % MVIC) [47]. In addition, the sit-up exercise reached ~0.41 mV [16].

Within the core stability exercises, the front plank with scapular adduction and a posterior pelvic tilt elicited the highest EMG activity (110.78 ± 65.76% MVIC) [26]. Furthermore, the front plank with additional weight (20% body weight) reached ~0.2 mV [28].

When the core exercises were performed on an additional ball/device, the greatest EMG activity levels were found during the stir-the-pot (144.20 ± 108.10% MVIC) and the front plank on a Swiss ball with hip extension (109.40 ± 65.20% MVIC) [32]. Regarding the studies reporting EMG activity as mV, the highest values were reached during the sit-up with upper and lower limb instability achieved by placing the feet and lower back on a BOSU (0.44 ± 0.22 mV), or with only the lower back on the BOSU (0.42 ± 0.22 mV) [16].

The Bulgarian squat had the highest EMG activity (Stable: ~155% MVIC; Unstable: ~148% MVIC) in the free-weight exercise group [30]. Also, the standing unilateral dumbbell press achieved 0.4 mV in the EO [86].

### 3.6. Erector Spinae

Activation of the ES was greater in back extension exercises (~63% MVIC) than in the other exercises analyzed [25,68] in the traditional core exercise group. 

In addition, back extension exercises showed the greatest activation in core stability exercises, not only on the floor (~63% MVIC) but also on the bench (~56% MVIC) [25]. One study, reporting in mV, also registered the front plank with additional weight (20% BW) at 0.1 mV [28]. 

Regarding core exercises on a ball/device, the suspended bridge showed the highest % MVIC (61.51 ± 13.85%) [50]. Only one study from this category reported EMG activity for the ES as mV (the 5-min Shaper device: 0.99 ± 0.06 mV) [37].

In relation to free-weight exercises, the greatest activation was found on the deadlift (barbell deadlift: ~90% MVIC; hex bar deadlift: ~80% MVIC) and hip-thrust exercise (~85% MVIC) [46]. Also, the back squat performed until failure and 2RM deadlift reported the highest mV values (~0.35 mV) [28,40].

### 3.7. Lumbar Multifidus

Only one study analyzed MUL activation in traditional exercises, which showed the highest % MVIC in prone trunk extensions and leg extensions with active lumbopelvic control (~64% MVIC) [56]. Concerning MUL activation for core stability, the highest % MVIC were found during the bridge exercise and bird dog (with light loads on the active hand and leg) (~39%) [71,82]. The highest mV were observed in bird dog (0.86 ± 1.01 mV) [85]. 

When it came to core exercises on a ball/device, the front plank on a Swiss ball with hip extension achieved ~62% MVIC for the MUL [32]. Three studies were conducted looking at free-weight exercises [44,59,70], with the greatest EMG activity found in the 45% body weight bent-over row (~58.20% MVIC), the 75% bodyweight deadlift (~57.90% MVIC) and the 75% body weight back squat (~54.80% MVIC) [44]. 

### 3.8. Transversus Abdominis

We found three studies analyzing this muscle [24,36,85]. Two studies examined TA activation based on % MVIC, in which the side-lying lumbar setting on a sling exercise reported the highest activation (58.65 ± 6.99%) [36] followed by the static curl-up with hands behind the neck (40.70 ± 26.50%) [24]. Based on mV values, a third study examined TA activation during the bird dog exercise (2.63 ± 3.11 mV) [85].

## 4. Discussion

The aim of this study was to systematically review the current literature on the electromyographic activity of six core muscles during core physical fitness exercises. Most of the studies on core muscle activation (55/67) reported EMG activity as % MVIC, with one of the main findings being that the greatest activity in the RA, EO and ES muscles was found in free-weight exercises. The greatest IO activity was observed in core stability exercises while the greatest MUL activation was found in traditional exercises. However, there was a lack of research on TA activation during core physical fitness exercises and a lack of consistency between studies in terms of the methods applied to measure EMG activity. 

### 4.1. Rectus Abdominis

Free-weight exercises elicited the greatest EMG activity in the RA during the unstable Bulgarian squat (unilateral) and the regular back squat (bilateral) with six maximum repetitions [30]. The RA demand increased throughout the repetitions, suggesting that the difference between the Bulgarian squat and the regular back squat would increase as the muscle became fatigued [30]. The fact that these exercises achieved the highest EMG activity in the RA might be explained not only by the heavy weights leading to exhaustion but also to the biomechanics of these exercises themselves [30,88]. The trunk tilts forward during the squat phase to compensate for the hip moving further backwards and, consequently, the EMG activity increases [30,88]. 

Core exercises on a ball/device, such as the roll-out plank [19,29] and the suspended front plank [29] were also recommended for achieving high RA activation. Suspension training systems add instability to the exercise, potentially leading to increased EMG activity. Also, it is essential to highlight that EMG activity may vary depending on the type of suspension training system used. For example, a previous study showed that pulley-based suspension systems elicited the greatest RA activation [17]. This type of suspension system may require greater postural control and strength requirements to perform the exercise with the proper technique than other suspension systems [17]. It is also important to consider where the instability is added. For example, one study found that the greatest EMG activity was observed when adding instability with the BOSU, not only on the feet but also on the lower back during the sit-up exercise [16]. Since the RA is a trunk muscle, generating upper body instability would require greater activation to maintain postural control [17].

### 4.2. Internal Oblique

The front plank with scapular adduction and posterior pelvic tilt, which belongs to the core stability exercise group, may be recommended for developing IO activation [26]. This isometric exercise showed the greatest activation values in the IO, perhaps due to the influence of the thoracolumbar fascia [26]. The IO is attached to the thoracolumbar fascia, and this plays an essential role in the transmission of load from the trunk to the shoulder and the arm [26,89]. In addition, the climax laughter exercise showed the highest mV values (~0.11 mV) [31]. This exercise, whose IO EMG activity was significantly greater than in the crunch exercise, requires high levels of internal muscular control [31]. Consequently, it is recommended as a core stability exercise for IO activation as well as for its psychological and hormonal benefits [31].

Although only a few studies have examined IO activation in free-weight exercises [33,34,35], kettlebell swings with the “Kime” variant registered the greatest EMG activity. The “Kime phase” involves a muscular pulse at the top of the kettlebell swing that trains quick muscle activation and relaxation. However, the same study showed that the large shear compression load ratio on the lumbar spine during the swing phase might be a reason to consider this exercise contraindicated in people with spine shear load intolerance [33].

### 4.3. External Oblique

Free-weight exercises, such as the Bulgarian squat, had the highest EMG activity [30]. The fact that this unilateral exercise showed such EO activity could be explained by the aim of this trunk rotation muscle, which is to prevent lateral flexion [90]. The exercise requires one foot in front of the other, and the greater the axial distance between them, the lower the stabilizing effect of the parallel legs and the greater the EO activity in preventing lateral sway [30]. In addition, another unilateral exercise (the standing unilateral dumbbell press) had the greatest EMG activity reported as mV [86]. A similar conclusion was drawn from this study—that the results may be explained by the EO’s contralateral effect in stabilizing the core and postural sway when performing the exercise [86]. In consequence, one can conclude that free-weight exercises are recommended for EO activation, especially those performed unilaterally due to the increases in EO activity.

Another possibility suggested by this systematic review is the addition of a ball or device to the core exercises. For example, it suggested front planks on a Swiss ball with the variant of moving the forearms in a continuous clockwise fashion (stir-the-pot), or doing a hip extension while maintaining stability, as being very intense EO exercises [32]. Adding stability balls leads to increased EMG [45,60,81]. Likewise, other researchers have observed increases in EMG activity in the EO when adding suspension training systems or whole-body wobble boards to the front plank exercise [45,78]. Since these instability systems challenge both proximal stability and distal mobility, exercises such as the front plank on a Swiss ball or stir-the-pot may be considered useful inclusions to core-strengthening programs [32].

### 4.4. Erector Spinae

One of the novel findings of this systematic review was that free-weight exercises (e.g., deadlift, hip-thrust, or back squat) showed the greatest ES muscle activation [38,46]. In this regard, some researchers [74] recommend adding destabilizing bars to free-weight exercises because when used with heavy weights, these bars have been designed to make the lifting action harder. Therefore, these exercises may be recommended since a high motor unit recruitment of the posterior chain is required to maintain a neutral posture regardless of the load’s center of mass and its effect on torque [74]. 

In addition, exercises such as a back extension on the floor showed high ES muscle activation [68]. A previous study observed that the ES activity was significantly higher in the hyperextension phase of the movement compared to the other exercise phases [91]. Hence, the one-legged back extension, which is another variant of this exercise, increases EMG activity in the ES and may also be recommended as a core physical fitness exercise [51].

Very similar EMG activities were found in the ES muscle when performing suspended bridge exercises [50]. The bridge is a traditional core exercise, but the addition of suspension training systems increased the recruitment of the abdominal, hamstring, gluteal, and trunk extensor muscles [50]. However, not all the devices that add instability increase ES activation. For example, previous studies found that performing the bridge on a Swiss ball, or a whole-body wobble-board platform, did not increase the activation of this muscle [45,82]. In consequence, these results suggest that the ES contributes to spinal control while maintaining a specific body posture, regardless of the type of exercise [82]. 

### 4.5. Lumbar Multifidus

The highest % MVIC for this muscle was observed during prone trunk extensions and prone leg extensions with active lumbopelvic control [56]. However, the muscle activity data from this study showed that the posterior extensor chain was more active when applying active lumbopelvic control strategies, which decreased the lumbar hyperlordosis [56]. Therefore, the exercise (e.g., trunk extensions) required greater hip extension and thus, the muscle activity increased [56]. 

The front plank on a Swiss ball with the hip extension exercise, which is one of the core exercises on a ball/device, can be recommended for developing MUL activation, given it had one of the highest % MVIC for this muscle [32]. The exercise elicited greater EMG activity than the static front plank on the floor, which suggests that adding the ball achieved the required instability to increase the EMG [32]. The activation levels of this muscle were high (>60% MVIC) during the exercise, which is in line with the definition of previous researchers that the MUL is a “local stabilizer” providing stability to the pelvis when performing the hip extension movement [92]. Consequently, this exercise is strongly recommended for strengthening purposes, given the high activity level that was observed not only in the core muscles, but also in the chest and lower limb [32].

Although only three studies were carried out on free-weight exercises [44,59,70], similar EMG activity to the front plank on a Swiss ball with the hip extension exercise was found for the MUL muscle in the 45% body weight bent-over row, the 75% bodyweight deadlift, and the 75% body weight back-squat exercises [44]. These are multi-joint exercises in which the trunk tilts forward during the squat phase to compensate for the hip motion, and the load is moved through the sagittal plane perpendicular to the position of the trunk. This position requires the back muscles to resist the high torques, which might explain the EMG activity results [93]. Despite observing that the above-mentioned exercises elicited the greatest EMG activity of the MUL, it should be pointed out that this systematic review found only 16 studies that examined this muscle. 

### 4.6. Transversus Abdominis

The greatest activation of this muscle was reported with suspension training systems using the side-lying lumbar setting of the sling exercise [36]. This study explained that the sling exercise, which can be performed in the prone, supine, or side-lying positions, developed the activation of local trunk muscles, such as the TA and MUL [36]. Despite the higher levels of EMG activity of the TA during the side-lying position, the authors recommended prone and supine sling exercises for stabilizing the lumbar region, given its high local/global muscle ratio [36].

Core stability exercises have also been recommended for TA activation [73,85]. Specifically, this systematic review found that the bird dog elicited greater EMG activity in the TA than in the IO or MUL [85], which might be because the TA is a primary trunk stabilizer, which modulates intra-abdominal pressure, the tension of the thoracolumbar fascia, and the compression of sacroiliac joints [85]. However, the scarcity of research on this muscle was another finding of our systematic review; indeed, only two studies were found that analyzed this muscle [36,85]. This contradicts a previous systematic review, which cited 10 studies analyzing the TA [2]; nonetheless, a decrease in the recent studies examining this muscle’s activity has been found. Therefore, future studies are needed that evaluate the TA’s EMG activity during core physical fitness exercises.

### 4.7. Limitations of the Study

As in our case, previous systematic reviews found methodological limitations in the selected studies that limited the quantitative summarization of the findings [2,94]. For example, the method chosen for determining EMG activity is an important decision in the study design stage [95]. Our systematic review found that 12 out of the 67 studies did not report EMG activity as % MVIC. This lack of agreement between the methods used for reporting EMG activity was also observed in previous reviews [2,94]. We suggest that future studies use % MVIC, as this is considered a more individualized method for reporting EMG activity, which may also reduce the risk of bias when interpreting the results in systematic reviews. 

In addition, we found a lack of consistency in applying methods to measure EMG activity. The methods used varied depending on the study, although the most frequent ones were designed with three sets of different durations (5–10 s). This methodological issue has also been pointed out as the main concern for the interpretation of EMG activity and the potential risk of bias [94]. Therefore, future studies need to reduce these differences in the methodology applied [94]. Furthermore, the level of evidence of the included studies was mainly moderate, which suggests that more high-quality research is necessary in order to the reduce the risk of bias and draw solid conclusions about core muscle activity [2]. In addition, the fact that the studies were included only if the full text was available in English, which is considered the universal language of science, may be another limitation of the study. 

Our systematic review also only focused on healthy adults, whereas populations such as the elderly and low-back patients still need to be studied. Furthermore, it would be of interest to analyze the activation patterns in individuals with different body fat levels since this variable may also influence the EMG activity recorded [96], which may be a potential risk of bias. In this regard, none of the studies compared EMG activity between males and females. Also, the addition of kinematical parameters to the EMG analysis would provide a holistic approach for determining which exercises are recommended.

## 5. Practical Applications

This systematic review provided a selection of exercises for greater activation of each core muscle group based on four different types of exercise (traditional core exercises, stability exercises, core exercises on a ball/device, and free-weight exercises) to assist strength and conditioning coaches, as well as fitness professionals. For example, free-weight exercises, such as the unstable Bulgarian squat, the regular back squat, roll-out plank, and the suspended front plank are suggested for RA activation. The front plank with scapular adduction and the posterior pelvic tilt, which belongs to the core stability exercise group, can be recommended for developing IO activation. Climax laughter exercises and kettlebell swings with “Kime” could be another alternative for IO activation (although swing exercises may be contraindicated for people with spine shear load intolerance). With regard to EO, unilateral free-weight exercises, such as the Bulgarian squat or the standing unilateral dumbbell press are recommended. Likewise, the front plank on a Swiss ball with the variant of moving the forearms in a continuous clockwise fashion (stir-the-pot) or doing a hip extension while maintaining stability are alternative exercises for this purpose. When it comes to the ES, free-weight exercises (e.g., the deadlift, hip-thrust, or back squat), the back extension on the floor, or the variant one-legged back extension, along with suspended bridge exercises, significantly increase EMG activity. To increase MUL activation, we suggest trunk extensions (with active lumbopelvic control), the front plank on a Swiss ball with the hip extension exercise, and free-weight exercises, such as the 45% bodyweight bent-over row, the 75% bodyweight deadlift, and 75% body weight back-squat exercises. Even though the greatest activation of the TA was reported with suspension training systems using the side-lying lumbar setting in the sling exercise, we instead suggest prone and supine sling exercises in order to stabilize the lumbar region, given its high local/global muscle ratio.

## 6. Conclusions

This study systematically reviewed the current literature on the EMG activity in six core muscles during core physical fitness exercises. The greatest activity in the RA, EO, and ES muscles was found in free-weight exercises. The greatest IO activity was found in core stability exercises, while traditional exercises showed the greatest MUL activation. However, there was a lack of research on TA activation during core physical fitness exercises and a lack of consistency between studies when applying methods to measure EMG activity. In addition, the level of evidence of the included studies was mainly moderate, which suggests that more high-quality research is necessary in order to the reduce the risk of bias and draw solid conclusions about core muscle activity.

## Figures and Tables

**Table 1:** Characteristics of the included studies.

Reference	*n*	Gender	Age (Years Old)	Exercise Type	Muscle Tested	Method	Level of Evidence
Li et al. (2020) China [36]	16	8 male and 8 female	20.60 ± 0.20	Ball/device core exercise	RA, TA, MUL, ES	3 × 10 s	Moderate
Silva et al. (2020) Brazil [37]	15	Male	23.65 ± 4.49	Traditional core exercise, Ball/device core exercise	RA, EO, ES	5 reps	Moderate
Saeterbakken et al. (2019) Norway [38]	18	Female	24.10 ± 4.50	Free-weight, Ball/device core exercise	RA, EO, ES	3RM	Strong
Kim (2019) South Korea [39]	32	Male and female	22.70 ± 1.80	Ball/device core exercise	RA, IO, EO	3 × 5 s	Moderate
Kohiruimaki et al. (2019) Japan [35]	8	Male	22.10 ± 3.80	Free-weight, Ball/device core exercise	RA, IO, EO	3 sets until failure	Moderate
Andersen et al. (2019) Norway [40]	15	Male	23.20 ± 2.20	Free-weight, Ball/device core exercise	ES	2RM	Strong
Panhan et al. (2019) Brazil [41]	16	Female	27.60 ± 3.70	Core stability exercise	RA, IO, EO	8 reps	Moderate
Park & Park (2019) South Korea [42]	18	Female	21.60 ± 2.40	Core stability exercise, Ball/device core exercise	RA, IO, ES	2 × 16 s	Moderate
Park, Lim & Oh (2019) South Korea [43]	24	18 female and 6 male	22.33 ± 2.53	Ball/device core exercise	RA, IO, MUL, ES	3 × 5 s	Weak
Lane et al. (2019) United States [44]	13	Male	31.80 ± 5.70	Free-weight exercise	EO, MUL	10 reps	Weak
Biscarini et al. (2018) Italy [45]	16	11 male and 7 female	29	Core stability exercise, Ball/device core exercise	RA, IO, EO, MUL, ES	12 × 12 s	Moderate
Crommert et al. (2018) Sweden [24]	10	Female	26.00 ± 3.00	Traditional core exercise	RA, IO, EO, TA	3 × 3 s	Moderate
Andersen et al. (2018) Norway [46]	13	Male	21.90 ± 1.60	Free-weight exercise	ES	1RM	Moderate
Kim & Park (2018) South Korea [47]	20	Male	22.55 ± 1.85	Traditional core exercise	RA, IO, EO,	6 reps	Moderate
Khaiyat & Norris (2018) United Kingdom [48]	20	Female	20.10 ± 1.10	Traditional core exercise, Core stability exercise, Free-weight exercise	RA, ES	10 reps	Moderate
Youdas et al. (2018) United States [32]	26	13 male and 13 female	25.20 ± 4.70	Traditional core exercise, Ball/device core exercise	RA, IO, EO, MUL	10 s	Moderate
Van den Tillaar & Saeterbakken (2018) Norway [28]	12	Male	23.50 ± 2.60	Core stability exercise, Free-weight exercise	RA, EO, ES	Until failure	Moderate
Calatayud et al. (2017) Spain [29]	20	13 male and 7 female	20.00 ± 1.00	Core stability exercise, Ball/device core exercise	RA, EO, ES	5 s	Moderate
Lyons et al. (2017) United States [49]	14	Male	21.50 ± 2.03	Free-weight exercise	EO, ES	10RM	Strong
Harris et al. (2017) United States [50]	25	16 male and 9 female	27.24 ± 4.02	Core stability, Ball/device core exercise	RA, EO, ES	5 reps (push-ups and rowing) and 30 s (plank and bridge)	Moderate
Cortell-Tormo et al. (2017) Spain [26]	15	10 male and 5 female	24.35 ± 4.29	Core stability	RA, IO, EO, ES	3 × 10 s	Moderate
Schellenberg et al. (2017) Switzerland [51]	16	8 male and 8 female	26.30 ± 4.20	Traditional core exercise	RA, EO, ES	8 reps	Moderate
Cugliari & Boccia (2017) Italy [19]	17	Male	27.30 ± 2.40	Ball/device core exercise	RA, IO, EO, ES	3 × 6 s	Moderate
Calatayud et al. (2017) Spain [52]	20	13 male and 7 female	20.00 ± 1.00	Traditional core exercise, Ball/device core exercise	RA, EO, ES	5 s	Moderate
Silva et al. (2017) Brazil [53]	15	Male	26.00 ± 5.00	Free-weight exercise	ES	10RM	Strong
Lee et al. (2017) South Korea [54]	7	6 male and 1 female	22.6 ± 2.23	Core stability, Ball/device core exercise	RA, EO, ES	10 s	Weak
Krommes et al. (2017) Denmark [55]	21	Male	21.40 ± 3.30	Core stability, Ball/device core exercise	RA, EO	3 × 6 s	Moderate
Van Oosterwijck et al. (2017) Belgium [56]	13	9 female and 4 male	22.60 ± 2.10	Traditional core exercise	MUL	10 reps	Weak
Kim et al. (2016) South Korea [57]	20	Male	30.44 ± 2.65	Core stability	RA, IO, EO	3 × 5 s	Weak
Kim & Lee (2016) South Korea [58]	20	12 female and 8 male	20	Traditional core exercise	RA, EO	5 × 3 s	Moderate
Youdas et al. (2016) United States [59]	26	13 male and 13 female	23.95 ± 2.64	Free-weight exercise	RA, MUL	3 ×10 s	Moderate
Escamilla et al. (2016) United States [60]	16	8 male and 8 female	27.70 ± 7.70	Core stability, Ball/device core exercise	RA, IO, EO	5 × 3 s	Moderate
Mello et al. (2016) Brazil [61]	19	Male	22.73 ± 11.28	Ball/device core exercise	MUL, ES	1 minute	Weak
De Blaiser et al. (2016) Belgium [62]	30	15 female and 14 male	25.50 ± 2.10	Core stability	RA, IO, EO, MUL	Until failure	Moderate
Andersen et al. (2016) Norway [63]	16	Male	25.00 ± 6.00	Free-weight exercise	RA, EO, ES	10 reps	Strong
Youdas et al. (2015) United States [64]	26	13 male and 13 female	23.45 ± 1.25	Traditional core exercise, Ball/device core exercise	MUL	3 reps	Moderate
Calatayud et al. (2015) Spain [65]	21	Male	25.00 ± 2.66	Free-weight exercise	EO, ES	3 reps	Moderate
Mok et al. (2015) China [66]	18	10 female and 8 male	21.90 ± 1.70	Core stability, Ball/device core exercise	RA, EO, MUL	5 reps	Weak
Ha et al. (2015) South Korea [67]	13	Male	39.00 ± 6.50	Core stability, Ball/device core exercise	RA, IO, EO	3 × 5 s	Moderate
Park et al. (2015) South Korea [68]	16	Male	23.00 ± 1.92	Traditional core exercise, Free-weight	ES	3 × 5 s	Strong
Yavuz et al. (2015) Cyprus [69]	12	Male	21.20 ± 1.90	Free-weight exercise	ES	1RM	Moderate
Borreani et al. (2015) Spain [70]	30	Male	23.00 ± 1.13	Free-weight exercise, Ball/device core exercise	MUL	5 reps	Strong
Masaki et al. (2015) Japan [71]	17	Male	22.40 ± 1.30	Core stability	MUL, ES	3 × 3 s	Moderate
Patterson et al. (2015) United States [34]	22	15 males and 7 females	22	Free-weight	IO, EO	5 reps	Moderate
Moon et al. (2015) South Korea [72]	10	Female	26.50 ± 4.22	Core stability	RA, EO	3 × 5 s	Moderate
Calatayud et al. (2014) Spain [17]	29	Male	23.50 ± 3.10	Free-weight, Ball/device core exercise	RA, ES	3 reps	Strong
Saeterbakken et al. (2014) Norway [16]	24	Male	23.00 ± 2.00	Traditional core exercise, Ball/device core exercise	RA, EO	10RM	Strong
Badiuk et al. (2014) Canada [73]	8	Male	22.50 ± 2.20	Core stability	RA, IO, EO	3 × 2 reps	Strong
Fletcher & Bagley (2014) [74] United Kingdom	14	Male	21.70 ± 2.60	Free-weight exercise	ES	1RM	Strong
Serner et al. (2014) Denmark [75]	40	Male	21.40 ± 3.30	Core stability	RA, EO	2 × 6 s	Strong
Van den Tillaar & Saeterbakken (2014) Norway [76]	14	Male	22.50 ± 2.00	Free-weight	RA, EO, ES	6RM	Moderate
Saeterbakken et al. (2014) Norway [77]	25	Female	24.30 ± 4.90	Free-weight	RA, EO, ES	6RM	Strong
Byrne et al. (2014) Canada [78]	21	11 male and 10 female	21.90 ± 2.40	Core stability, Ball/device core exercise	RA, EO	2 × 3 s	Moderate
Calatayud et al. (2014) Spain [79]	29	Male	22.6 ± 2.6	Free-weight, Ball/device core exercise	RA, EO	3 reps	Strong
Calatayud et al. (2014) Spain [80]	29	Male	23.50 ± 3.10	Free-weight, Ball/device core exercise	RA, ES	3 reps	Strong
Wagner et al. (2014) Germany [31]	14	7 male and 7 female	21.50 ± 1.30	Traditional core exercise, Core stability exercise	RA, IO, EO	3 × 10 s	Weak
Ishida & Watanabe (2014) Japan [27]	12	Male	21.20 ± 2.80	Core stability	RA, IO, EO	3 × 5 s	Weak
Andersen et al. (2014) Norway [30]	15	Male	24.00 ± 4.00	Free-weight	RA, EO, ES	6RM	Strong
Czaprowski et al. (2014) Poland [81]	33	18 female and 15 male	23.20 ± 2.50	Core stability, Ball/device core exercise	RA, EO	3 × 5 s	Moderate
Kim et al. (2013) South Korea [82]	20	Male	23.35 ± 2.01	Core stability, Ball/device core exercise	RA, IO, MUL, ES	3 × 10 s	Moderate
Saeterbakken et al. (2013) Norway [83]	15	Male	23.30 ± 2.70	Free-weight, Ball/device core exercise	RA, EO, ES	3 s	Strong
Saeterbakken et al. (2013) Norway [84]	16	Male	22.50 ± 2.00	Free-weight, Ball/device core exercise	RA, EO, ES	6RM	Strong
Maeo et al. (2013) Japan [25]	10	Male	21.20 ± 1.50	Traditional core exercise, Core stability exercise	RA, IO, EO, ES	10 s/10 reps	Moderate
Pirouzi et al. (2013) Iran [85]	30	Female	23.13 ± 2.41	Core stability	IO, TA, MUL	3 × 5 s	Moderate
Saeterbakken & Steiro (2012) Norway [86]	15	Male	22.00 ± 2.00	Free-weight	RA, EO, ES	5 reps	Strong
Tarnanen et al. (2012) Finland [87]	20	Female	38.10 ± 7.00	Traditional core exercise, Ball/device core exercise	RA, EO, MUL	1RM	Strong
McGill & Masrshall (2012) United States [33]	7	Male	25.60 ± 3.40	Free-weight exercise	RA, IO, EO, ES	1 rep	Moderate

RA: rectus abdominis; IO: internal oblique; EO: external oblique; TA: transversus abdominis; MUL: lumbar multifidus; ES: erector spinae; RM, maximum repetition.

**Table 2:** Description of core physical fitness exercises assessed in the included studies.

**Curl-Up or Crunch**	Participant is in a supine position with feet on the floor (hip-width apart) and knee flexion. Upper body is lifted with hands behind the neck and then returned to the starting position. This exercise may vary depending on specific protocols (e.g., arms placed across the chest, 90° hip and knees flexion, curl-up with twist) [24,25,31,37,47,48].
**Side Crunch**	Participant is in a side-lying position with knees lightly flexed. Upper body is lifted with hands behind the neck and then returned to the starting position. This exercise may vary depending on specific protocols (e.g., arms placed across the chest, side crunch on unstable surfaces) [60,68].
**Sit-Up**	Participant is in a supine position with feet on the floor (hip-width apart) and knee flexion. Upper body is lifted with hands behind the neck and then returned to the starting position. The exercise is similar to the curl-up or crunch, but the main difference is that the aim of the sit-up is to lift the trunk until the participant is upright in a semi-seated position. This exercise may vary depending on specific protocols (e.g., sit-ups on unstable surfaces) [16,25,58,60].
**Roll-Up**	Participant is in a supine position with the posterior chain lying down on the floor. The participant is asked to breathe in and tuck the chin in toward the body. Then, the participant is asked to breathe out while rolling up and lifting the upper body [72].
**V-Sits**	Participant is in a supine position on the floor with the arms the head in addition to hip and knee extension. The participant is instructed to lift the legs through a hip flexion movement (45°) and lift the arms towards the ankles [25].
**Bilateral Leg Raise**	Participant is in a supine position with palms down on the floor. The participant raises both legs with a hip flexion movement while keeping the knees extended [42,48].
**Straight One-Leg Hold**	Participant is in a supine position with hands on the abdomen. One foot is lifted with hip flexion movement (straight one-leg hold, 45°). The supporting leg keeps knee flexion and one foot on the floor. This exercise may vary depending on specific protocols (e.g., foam roll or balance cushion as foot support) [39,67,72].
**Double Leg Stretch**	Participant is in a supine position, with the hip and knees flexed toward the core and the hands touching the patella. The exercise is performed with simultaneous upper and lower limb extension while the head, shoulders, arms, and legs remain off the supporting base [41].
**Leaning Forward**	Participant is in a kneeling position while hanging from a sling system with the proximal forearm leaning forward, as well as drawing in the lower abdomen after expiration (until 90° shoulder flexion) [43].
**Back Extension**	Participant is in a prone position, keeping the neutral position of the spine. The participant moves up and down. If the back extension is performed on a bench, the thighs are placed on a pad and the feet are fixed to the bench. This exercise is characterized by the force of gravity. This exercise may vary depending on specific protocols (e.g., one-legged back extension) [25,51,56,68,87].
**Prone Leg Extension**	Participant is in a prone position with the upper body strapped to the table at the scapula. The leg extension movement is performed by the bilateral flexion and extension of the hip [56].
**Bird Dog**	Participant is in a prone position with knees under the hips and hands under the shoulders. The participant raises the right arm with a 180° shoulder flexion and the left leg with hip extension. In consequence, if the left arm is raised, the right leg performs the hip extension. This exercise may vary depending on specific protocols (e.g., static or dynamic bird dog, bird dog with hip abduction, bird dog with loads) [45,71,85].
**Bridge**	Participant laid in a supine position with knees flexed (90°) and feet on the floor. The hip is lifted in a hip extension movement, keeping a straight line between the shoulders and the knees. This exercise may vary depending on specific protocols (e.g., suspended bridge, bridge with hamstring curl on Swiss ball, unilateral bridge or supine plank on suspension systems) [25,45,48,50,52,61,64,66,72,81,82].
**Front Plank**	Participant in prone position with posterior pelvic tilt and body weight supported by the forearms and feet. The feet are shoulder-width apart and the spine keeps its neutral position. This exercise may vary depending on specific protocols (e.g., front planks with hands and/or legs on suspension systems or unstable surfaces) [25,26,28,29,32,42,45,50,54,57,60,62,66,78,81].
**Lateral Plank**	Participant in side-lying position with the elbow beneath the shoulder, making a 90° angle (forearm is placed on the floor). The hip is lifted with spine in its neutral position and knees extended in order to keep a straight line from the head to the feet. This exercise may vary depending on specific protocols (e.g., suspended lateral plank, lateral plank on knees, remove the forearm from the floor and keep elbows extended) [25,27,29,45,60,81].
**Stable Roll-Out Plank**	Participant is in a prone position with feet, knees, and hands on the floor. The participant rolls out with the elbows extended until the hip and knees are aligned. This exercise may vary depending on specific protocols (e.g., suspended roll-out plank) [19,29].
**Abdominal Drawing-In Maneuver**	Participant is in a supine position with the knees flexed and feet on the floor. This exercises increases the abdominal pressure by pulling the abdominal walls to the inside [72].
**Climax Laughter**	Participant in standing position with feet on the floor. The participant starts to laugh after a deep intake of breath [31].
**Bracing**	Participant is in a supine position with knees flexed. The participant is instructed to contract the abdominal muscles followed by two breaths while keeping the contraction [25].
**Hollowing**	Participant is in a supine position with knees flexed. The participant is instructed to draw in the abdominal muscle towards the spine [25,73].
**Lewit**	Participant is in a supine position with the hip and knees flexed (90°). The participant is instructed to follow a regular breath pattern. Then, the participant is instructed to purse the lips as if breathing through a straw. Full effort is required to expel the air [73].
**Maximum Expiration**	The participant, who is in a supine position, is instructed to hold the breath after maximum expiration with an open airway [27].
**Stir-The-Pot**	Participant is in a prone position with feet on the floor and elbows under the shoulder and the forearm on a Swiss ball. The participant continually moves forearms in a clockwise manner while keeping the spine in its neutral position [32].
**Suspended Pike**	Participant is in a prone position similar to a front plank on a suspension system (with hands on the floor and feet suspended). The hip is flexed (90°) while keeping the knees fully extended [19].
**Suspended Body Saw**	Participant is in a prone position similar to a front plank on a suspension system (with hands on the floor and feet suspended). The shoulders are flexed and the elbows are extended in order to push the body forward and backward [19].
**Suspended Knee-Tuck**	Participant is in a prone position similar to a front plank on a suspension system (with hands on the floor and feet suspended). The elbows are extended, the hips and knees are flexed (90°) in order to move the knees forward and backward [19].
**5-min Shaper Device**	Participant in prone position with hands on a handlebar. The knees are placed on a supporting surface of the device in order to perform hip flexion and extension. The angle of the device relative to the ground may be modified based on four levels of intensity (beginner, intermediate, advanced, and extreme) [37].
**Supine Lumbar Setting on Sling**	Participant is in a supine position with arms on the abdomen, hip flexion (90°), and knee flexion (90°). The legs hang from a sling [36].
**Side-Lying Lumbar Setting on Sling**	Participant is in a side-lying position with the head, chest, and legs hanging from slings [36].
**Prone Lumbar Setting on Sling**	Participant is in a prone position with the head, chest, and legs hanging from slings [36].
**Copenhagen Adduction**	Participant is in a side-lying position with one forearm (e.g., right forearm) as support on the floor. The leg on the forearm side (i.e., right leg) is also on the floor, providing stability to the body through the ankle. The other leg is held by another partner at its hip’s height. The participant is instructed to perform a hip adduction and then return to the starting position [75].
**Hip Adduction with an Elastic Band**	Participant is in a standing position with an elastic band on the leg, which is placed closer to the band’s fixation point. The aim is to adduct the hip while maintaining balance with the other foot [75].
**Hip Adduction on an Adductor Machine**	Participant is in a sitting position on a hip adduction machine. The participant has to adduct the hip since the machine adds resistance towards maximal hip abduction [75].
**Hip Adduction against a Partner’s Hip Abduction**	This exercise requires two participants who are sitting on the floor. Both of them have their hands placed on the floor behind the trunk. The tested participant places the legs with the knees extended and the feet and lower shin on the outside (i.e., distally) of the partner’s feet and lower shin while performing an adduction, which is balanced by the partner’s abduction [55].
**Hip Abduction against a Partner’s Hip Adduction**	This exercise requires two participants who are sitting on the floor. Both of them have their hands placed on the floor behind the trunk. The tested participant places the legs with the knees extended and the feet and lower shin medially on the partner’s feet and lower shin while performing an abduction, which is balanced by the partner’s adduction [55].
**Sliding Hip Abduction/Adduction Exercise**	Participant is in a standing position with hands on the hip. One foot is placed on a washcloth in order to slide it laterally by abducting the hip; it is then returned to the starting position [75].
**Side-Lying Hip Adduction**	Participant is in a side-lying position, with the side-lying leg (e.g., right leg) straight and the left leg with hip and knee flexion (90°). The aim is to lift the right leg by hip adduction [75].
**Supine Hip Adduction**	Participant is in a supine position with the hip and the knees in 90° flexion. The aim is to perform maximal hip abductions and return to the starting position by hip adduction [75].
**Isometric Ankle Adduction against a Ball**	Participant is in a supine position with knees extended. The participant has to press against a ball, which is placed between the ankle [55,75].
**Isometric Knee Adduction against a Ball**	Participant is in a supine position with knees flexed. The participant has to press against a ball, which is placed between the knees [55,75].
**Standing One-Leg Cross-Country Skiing**	Participant is in a unilateral standing position. It is a coordination exercise in which the knee is continually flexed and extended while swinging the arms [55].
**Folding Knife**	Participant is in a supine position with a ball between the knees. The repetitions are performed by the participant combining a sit-up with hip and knee flexion [55].
**Forward Lunge**	Participant is in a standing position with feet shoulder-width apart. One step forward is taken in the sagittal plane. The participant lowers the body (spine in neutral position) with 90° hip and knee flexion [48].
**Push-Up**	Participant is in a prone position with shoulders abducted and elbows extended. The toes are placed shoulder-width apart. When flexing the elbows, the participant lowers the body and pushes back up by extending the elbows. This exercise may vary depending on specific protocols (e.g., push-ups on suspension systems with hands and/or legs on the suspension system, push-ups with hands and/or legs at different heights) [17,35,50,70,79,80].
**Back Squat**	Participant is in a standing position with fully extended knees and a natural sway in the lower back. The barbell is placed behind the neck. The participant lowers the load (using a self-paced but controlled tempo) such that the fulcrum of the hip is equal to the fulcrum of the knees (full squat). All parts of the feet are in contact with the floor. This exercise may vary depending on specific protocols (e.g., back squat on unstable surfaces, on Smith machine, different loads, or half-squat) [28,30,38,44,48,53,69,74,77,83].
**Bulgarian Squat**	Participant is in a standing position with feet on the floor. One step forward is taken with the front leg and the other one in the back is placed on a bench. The distance between the bench and the front leg is ~80% of the leg length. Then, the barbell is placed behind the neck. The aim of the exercise is to lower the load by squatting with the front leg [30].
**Front Squat**	Participant is in a standing position with fully extended knees and a natural sway in the lower back. The barbell is placed behind the neck. The movement is similar to the back squat but the main difference is that the barbell is placed across the front side of the shoulders [69].
**Deadlift**	The participant, in a standing position with feet shoulder-width apart, has to lower the body in order to lift the barbell until the hip is fully extended while maintaining a neutral spine position. This exercise may vary depending on specific protocols (e.g., Romanian deadlift, stiff leg, hexagonal bar deadlift) [40,44,46].
**Hip Thrust**	Participant is in a seated position on the floor with the upper back on a bench and knees flexed at 90°. The barbell is placed above the pelvis and the spine is maintained in a neutral position. The aim is to lift the barbell with a hip extension movement (i.e., hip thrust) [46].
**Kettlebell Swing**	Participant is in a squatting position with one hand (e.g., right hand) holding a kettlebell. The participant moves the kettlebell in the sagittal plane by rapidly extending the knees and hip until reaching the chest level. This exercise may vary depending on specific protocols (e.g., standing position as starting position, bilateral swings) [33,49,63].
**Kettlebell Snatch**	Participant in squatting position with one hand (e.g., right hand) holding a kettlebell (similar to the swing technique). The participant swings the kettlebell into a snatch position and the kettlebell is caught overhead. The force is absorbed by flexing the knees and hip as the participant performs the catch. In addition, the participant is told to keep the elbow extended (not locked) and hold the kettlebell overhead for a few seconds (e.g., two seconds) before going back to the starting position. This exercise may vary depending on specific protocols (e.g., standing position as starting and ending position, swings overhead) [33,49].
**Kettlebell Clean**	Participant in squatting position with the feet slightly wider than the shoulders. The participant reaches down to grasp the kettlebell with one hand (e.g., right hand) and pulls it up close to the body so that the elbow is high with shoulder abduction and elbow flexion. Once the bell is pulled high, the elbow and hand drop while the shoulder is performing external rotation. The kettlebell flips over the hand and it is caught posterior to the vertical forearm. Then, the participant absorbs the force by knee and hip flexion [49].
**Clean and Jerk**	The participant is in a standing position with feet shoulder-width apart has to lower the body in a squatting position in order to grasp the barbell with both hands. The participant pulls the barbell up (as much as possible) close to the body in addition to tripling lower limb extension (ankle, knee, and hip). Then, the barbell is received in front of the neck (resting on the shoulders) while getting into a squat position. Finally, the bar is lifted upwards while keeping the torso erect, feet flat on the floor, and the bar slightly behind the head [65].
**Bent-Over Row**	Participant is in a standing position with feet shoulder-width apart. Although the knees are flexed in order to lean the trunk forward from the waist, the spine remains neutral. The hands hold the bar slightly wider than shoulder-width apart with elbows extended. The aim of the exercise is to row the weight up until it touches the upper part of the abdomen [44].
**Inverted Row**	Participant is in a supine position with heels on the floor. The participant holds a barbell wider than shoulder width and pulls up in order to continually bring the chest to the barbell. This exercise may vary depending on specific protocols (e.g., suspended inverted row, inverted row with pronated grip) [50,59,66,68].
**Kneeling Rotational Throw**	Participant is in a short lunge position (one knee on the ground). Knees (hip-width apart) and shoulders remain in the same direction (perpendicular to the target: wall or partner). The aim is to perform a rotational throw with a heavy ball [44].
**Bench Press**	Participant is in a supine position with feet on the floor and spine in its neutral position on a higher surface (i.e., bench). The hands grasp the barbell wider than shoulder width and the participant lowers it to the chest. Once the barbell touches the chest, the barbell is pushed upwards until the elbows are extended. This exercise may vary depending on specific protocols (e.g., bench press on unstable surfaces, unilateral bench press) [34,76,79,84].
**Standing Cable Press**	Participant is in a standing position in the center of the pulleys with the feet shoulder-width apart. Once the spine is in its neutral position, the scapula is retracted, the elbows are flexed (90°), the shoulders abducted (45°), and the handles are moved forward by extending the elbow [79].
**Chest Press on Suspension Device**	Participant is in a prone position with heels on the floor, hips and knees extended. The participant holds onto the straps of the suspension system in order to perform a similar movement to push-ups by continually flexing and extending the elbows while maintaining the neutral position of the spine [66].
**Dumbbell Press**	Participant is in a sitting position with the feet shoulder-width apart and the knees flexed (90°, seated dumbbell press) or extended (standing dumbbell press). The participant holds the dumbbells with the thumb-side towards the ears. The aim of the exercise is to press the dumbbells straight forward while the spine maintains its neutral position. When doing the seated dumbbell press, the bench supports the back of the participant. This exercise may vary depending on specific protocols (e.g., unilateral seated dumbbell press, unilateral standing dumbbell press) [86].
**Triceps Dips**	Participant is in a sitting position with hands on push-up handles and feet elevated. The aim of the exercise is to lower the body by flexing the elbows and then lifting the body again [68].

°: degrees.

**Table 3:** Descriptive statistics of electromyographic activity (expressed as percentage of maximum voluntary contraction, % MVIC) in each study by exercise.

Reference	Core Exercise	RA	IO	EO	TA	MUL	ES	Conclusion
Li et al. (2020) [36]	Supine lumbar setting on a sling	20.66 ± 3.63			20.08 ± 2.77	42.52 ± 11.13	20.08 ± 2.77	Sling exercises could be effective exercises to enhance MUL and TA EMG activity. Specifically, supine and prone exercises were recommended in order to stabilize the lumbar region, given its high local/global muscle ratio.
	Prone lumbar setting on a sling	24.10 ± 3.86			55.51 ± 0.66	39.55 ± 6.58	17.89 ± 2.63	
	Left side-lying lumbar setting on a sling	19.00 ± 3.09			55.93 ± 6.42	36.77 ± 3.31	36.68 ± 3.97	
	Right side-lying lumbar setting on a sling	19.84 ± 3.42			58.64 ± 6.99	45.03 ± 5.10	33.68 ± 3.55	
Silva et al. (2020) [37]	Crunch	Upper: 21.47 ± 3.19 Lower: 14.65 ± 1.86		9.01 ± 1.13			0.86 ± 0.05	Greater activation was found in RA for all the exercises. Upper RA activation was greater than lower RA activation. Crunch elicited greater or similar EMG activity than exercises performed with the 5-min Shaper device. This device could be used in order to achieve variation between exercises. However, both exercises generate low abdominal muscle activation (<20% MVIC). Therefore, these exercises could be used for muscle endurance training.
	5-min Shaper device beginner	Upper: 10.64 ± 2.25 Lower: 8.61 ± 1.01		5.42 ± 0.74			0.72 ± 0.07	
	5-min Shaper device intermediate	Upper: 13.79 ± 2.91 Lower: 11.44 ± 1.48		6.98 ± 1.06			0.82 ± 0.08	
	5-min Shaper device advanced	Upper: 18.35 ± 3.25 Lower: 13.50 ± 1.64		8.09 ± 1.24			0.89 ± 0.05	
	5-min Shaper device extreme	Upper: 21.79 ± 4.38 Lower: 17.24 ± 1.53		9.55 ± 1.34			0.99 ± 0.06	
Saeterbakken et al. (2019) [38]	Back squat	~17		~20			~75	There were no significant differences in EMG activity between both exercises.
	Back squat on Smith machine	~15		~17			~75	
Kim (2019) [39]	Straight one-leg hold (45°)	19.10 ± 14.98	17.36 ± 9.50	22.13 ± 13.64				Greater EMG activity was observed when adding upper-body and lower-body instability.
	Straight one-leg hold (45°) with the foot on a balance cushion	20.99 ± 12.62	16.35 ± 8.43	26.38 ± 17.29				
	Straight one-leg hold (45°) with the low back on a foam roll	29.95 ± 12.85	23.90 ± 15.47	36.56 ± 26.88				
	Straight one-leg hold (45°) with the foot on a balance cushion and low back on a foam roll	32.55 ± 17.33	23.14 ± 13.84	38.41 ± 25.40				
Kohiruimaki et al. (2019) [35]	Suspended push-up	~80	~45	~45				Suspended push-up showed greater RA activity than EO and IO.
Panhan et al. (2019) [41]	Double leg stretch on mat	~55	~57					The short box significantly increased the EMG activity.
	Double leg stretch on long box	~66	~71					
	Double leg stretch on short box	~84	~92					
Park & Park (2019) [42]	Front plank	51.83 ± 17.44	45.78 ± 16.16				23.42 ± 7.22	Front plank and bilateral leg raise exercises similarly activate trunk musculature. Greater activation of IO and RA than ES was found in all the exercises.
	Front plank with a horizontal level	58.99 ± 15.19	52.89 ± 20.42				26.38 ± 9.21	
	Bilateral leg raise	63.79 ± 16.95	47.21 ± 12.71				17.77 ± 8.14	
	Bilateral leg raise with a horizontal level	65.82 ± 18.90	52.97 ± 14.33				21.25 ± 7.95	
Park, Lim & Oh (2019) [43]	Leaning forward alone	24.73 ± 18.58	21.40 ± 11.82			8.73 ± 9.43	5.47 ± 2.26	The integration of shoulder movements during leaning-forward exercises could be effective in the facilitation of the EMG activity of IO and MF muscles, especially with shoulder flexion.
	Leaning forward with horizontal shoulder abduction	26.86 ± 15.65	30.36 ± 15.68			13.81 ± 17.78	6.23 ± 3.35	
	Leaning forward with shoulder flexion	39.11 ± 22.12	40.35 ± 22.85			13.47 ± 14.17	5.85 ± 1.78	
Lane et al. (2019) [44]	75% BW Back Squat			10.90		54.80		The greatest activation was found in MUL during 45% BW Bent-over row. However, the greatest EO activation was observed during a kneeling rotational throw. These exercises developed greater EMG activity in MUL than EO.
	75% BW Romanian deadlift			10.10		57.90		
	45% BW Bent-over row			8.2		58.20		
	6 kg ball - Kneeling rotational throw			20.2		31.90		
Biscarini et al. (2018) [45]	Front plank	15.30 ± 7.89	16.60 ± 12.56	19.20 ± 8.86		4.00 ± 2.75	2.70 ± 0.72	Core exercises on whole-body wobble board increased EMG activity, avoiding the addition of external loads.
	Side plank	18.10 ± 9.51	16.80 ± 6.39	31.80 ± 10.61		15.20 ± 9.41	23.30 ± 13.25	
	Bridge	3.60 ± 2.30	4.30 ± 2.70	1.90 ± 1.10		27.20 ± 12.50	27.10 ± 9.30	
	Supine position with hip at 90°	14.20 ± 6.17	15.90 ± 8.10	22.70 ± 8.66				
	Bird-dog	4.90 ± 3.24	11.10 ± 7.10	16.60 ± 6.09		25.80 ± 10.91	21.80 ± 8.37	
	Front-plank on whole-body wobble board	19.90 ± 11.65	19.90 ± 9.69	29.80 ± 8.90		4.90 ± 3.47	3.10 ± 1.07	
	Side-plank on whole-body wobble board	25.40 ± 11.50	27.70 ± 11.90	41.60 ± 15.70		18.10 ± 13.00	27.20 ± 19.30	
	Bridge on whole-body wobble board	6.40 ± 4.60	17.2 ± 11.00	6.20 ± 3.80		26.30 ± 9.10	26.60 ± 8.60	
	Supine position with hip at 90° on whole-body wobble board	15.80 ± 5.09	21.70 ± 9.71	29.90 ± 12.86				
	Bird dog on whole-body wobble board	8.10 ± 4.90	10.80 ± 5.10	26.60 ± 8.40		18.30 ± 8.50	16.40 ± 6.60	
Crommert et al. (2018) [24]	Curl-up with straight arms in front (static)	60.80 ± 16.20	43.50 ± 9.10	31.40 ± 17.90	21.10 ± 17.10			The greatest EMG activity was elicited by the curl-up with hands behind the neck. Static positions also show greater values than dynamic positions.
	Curl-up with arms crossed over chest (static)	67.60 ± 15.70	47.10 ± 10.40	40.20 ± 21.90	21.50 ± 21.00			
	Curl-up with hands behind the neck (static)	81.00 ± 10.90	61.70 ± 17.00	58.80 ± 22.60	40.70 ± 26.50			
	Curl-up with twist (static)	52.20 ± 13.50	57.30 ± 12.40	48.90 ± 20.60	34.50 ± 24.80			
	Curl-up with straight arms in front (up phase)	43.70 ± 16.70	28.80 ± 10.00	13.00 ± 6.90	13.20 ± 8.80			
	Curl-up with arms crossed over chest (up phase)	49.00 ± 15.90	33.00 ± 11.30	17.20 ± 10.50	14.70 ± 16.90			
	Curl-up with hands behind the neck (up phase)	62.90 ± 13.90	49.90 ± 8.80	30.70 ± 17.30	28.90 ± 19.30			
	Curl-up with twist (up phase)	36.90 ± 13.00	44.70 ± 14.40	22.30 ± 10.90	26.30 ± 25.20			
Andersen et al. (2018) [46]	Deadlift						~90	No differences were found in ES activation between the exercises.
	Hex bar deadlift						~80	
	Hip thrust						~85	
Kim & Park (2018) [47]	Curl-up with 45° hip flexion	49.36 ± 14.51;	36.92 ± 18.68	50.61 ± 14.37				Curl-up with hip flexion at 90° increased EMG activities of IO and EO.
	Curl-up with 90° hip flexion	50.77 ± 16.45	48.67 ± 12.22	65.18 ± 24.83				
Khaiyat & Norris (2018) [48]	Double leg raise	43.30 ± 4.40					9.50 ± 2.20	Great activation was elicited in RA during the double leg raise while the squat showed the greatest activation of ES.
	Forward lunge	6.90 ± 0.09					11.10 ± 1.60	
	Bridge	4.80 ± 0.80					22.80 ± 2.90	
	Curl-up	36.60 ± 4.70					16.70 ± 3.70	
	Squat	9.20 ± 5.40					40.40 ± 18.30	
Youdas et al. (2017) [32]	Front plank	41.20 ± 24.60	58.30 ± 38.60	76.40 ± 63.40		24.60 ± 27.10		RA, IO, EO, and MUL significantly increased during the stir-the-pot and front plank on a Swiss ball with hip extension compared to the rest of the exercises.
	Front plank on a Swiss ball	54.70 ± 31.60	64.90 ± 49.10	88.30 ± 56.00		22.00 ± 27.50		
	Stir-the-pot	71.80 ± 35.70	73.50 ± 31.30	144.20 ± 108.10		27.80 ± 27.00		
	Front plank on a Swiss ball with hip extension	55.70 ± 26.20	76.50 ± 37.00	109.40 ± 65.20		62.20 ± 50.20		
Calatayud et al. (2017) [29]	Front plank	Upper: 32.00 ± 4.00 Lower: 30.00 ± 4.00		37.00 ± 5.00			2.00 ± 1.00	Suspended front and roll-out plank developed greater RA (even greater activation on the upper RA) and EO activity than the rest of the exercises. Lateral plank and suspended lateral plank were the exercises with the highest ES activation.
	Suspended front plank	Upper: 131.00 ± 15.00 Lower: 93.00 ± 10.00		88.00 ± 14.00			4.00 ± 1.00	
	Lateral plank	Upper: 26.00 ± 4.00 Lower: 20.00 ± 3.00		62.00 ± 12.00			13.00 ± 1.00	
	Suspended lateral plank	Upper: 31.00 ± 5.00 Lower: 30.00 ± 4.00		74.00 ± 14.00			14.00 ± 2.00	
	Unilateral front plank	Upper: 30.00 ± 5.00 Lower: 37.00 ± 8.00		44.00 ± 9.00			2.00 ± 1.00	
	Unilateral suspended front plank	Upper: 43.00 ± 9.00 Lower: 37.00 ± 8.00		58.00 ± 11.00			3.00 ± 1.00	
	Stable roll-out plank	Upper: 100.00 ± 12.00 Lower: 74.00 ± 11.00		49.00 ± 7.00			2.00 ± 1.00	
	Suspended roll-out plank	Upper: 145.00 ± 22.00 Lower: 122.00 ± 32.00		84.00 ± 12.00			4.00 ± 1.00	
Lyons et al. (2017) [49]	Kettlebell swing			15.60 ± 6.00			60.90 ± 24.30	These exercises increased ES activity. Specifically, kettlebell swings elicited the greatest activation of ES. However, kettlebell clean and snatch showed greater EO activation than kettlebell swings.
	Kettlebell snatch			20.70 ± 7.70			38.40 ± 17.70	
	Kettlebell clean			23.40 ± 10.10			51.00 ± 18.40	
Harris et al. (2017) [50]	Front plank	74.94 ± 30.26		54.63 ± 23.25			41.28 ± 23.33	The use of suspension systems increased EMG activity in RA, EO, and ES. The greatest RA activation was found in the suspended front plank, while the suspended push-up elicited the greatest EO activation, and the suspended bridge elicited the greatest ES activation.
	Suspended front plank	121.09 ± 118.98		66.79 ± 24.12			40.67 ± 16.45	
	Push-up	67.47 ± 25.26		52.80 ± 26.74			41.10 ± 15.96	
	Suspended push-up	93.90 ± 36.70		81.04 ± 60.14			54.52 ± 21.96	
	Inverted row	63.43 ± 18.94		37.44 ± 20.39				
	Suspended inverted row	67.41 ± 21.27		40.56 ± 24.74				
	Bridge	57.73 ± 15.77		33.11 ± 12.09			45.50 ± 9.47	
	Suspended bridge	60.83 ± 15.60		41.44 ± 28.01			61.51 ± 13.85	
Cortell-Tormo et al. (2017) [26]	Front plank with scapular abduction and anterior pelvic tilt	38.36 ± 25.69	49.76 ± 24.02	35.05 ± 29.95			4.74 ± 1.48	Posterior pelvic tilt elicited greater RA, EO, and ES activation, particularly when adding scapular adduction.
	Front plank with scapular abduction and posterior pelvic tilt	53.29 ± 19.54	70.43 ± 35.46	73.53 ± 31.11			5.48 ± 2.14	
	Front plank with scapular adduction and anterior pelvic tilt	33.56 ± 34.31	48.27 ± 29.72	40.26 ± 29.72			5.56 ± 1.73	
	Front plank with scapular adduction and posterior pelvic tilt	77.48 ± 43.72	119.92 ± 60.28	110.78 ± 65.76			7.43 ± 2.10	
Schellenberg et al. (2017) [51]	One-legged back extension	2.70 ± 3.90		4.60 ± 5.30			22.80 ± 8.20	One-legged back extension elicited greater muscle activation than a two-legged back extension. ES showed greater activation than RA and EO during these exercises.
	Two-legged back extension	2.10 ± 2.30		4.30 ± 4.60			18.90 ± 7.60	
Cugliari & Boccia (2017) [19]	Suspended pike	Upper: 41 ± 48 Lower: 57 ± 36	23 ± 20	55 ± 21			Upper: 9 ± 4 Lower: 12 ± 7	Suspended roll-out plank elicited the greatest activation in RA, IO, EO, and ES.
	Suspended body saw	Upper: 57 ± 52 Lower: 100 ± 42	32 ± 20	59 ± 33			Upper: 8 ± 6 Lower: 4 ± 3	
	Suspended knee-tuck	Upper: 44 ± 41 Lower: 54 ± 50	18 ± 26	42 ± 7			Upper: 6 ± 5 Lower: 8 ± 5	
	Suspended roll-out plank	Upper: 67 ± 78 Lower: 140 ± 89	40 ± 31	71 ± 44			Upper: 11 ± 6 Lower: 9 ± 5	
Calatayud et al. (2017) [52]	Suspended supine plank	Upper: 12.00 ± 9.00 Lower: 7.00 ± 10.00		11.00 ± 9.00			16.00 ± 1.00	Unilateral suspended plank elicited the greatest EMG activity in RA, EO, and ES. There were no significant differences between conditions for EO or upper or lower RA.
	Supine plank	Upper: 5.00 ± 9.00 Lower: 1.00 ± 11.00		5.00 ± 9.00			11.00 ± 1.00	
	Unilateral suspended plank	Upper: 15.00 ± 9.00 Lower: 11.00 ± 11.00		15.00 ± 9.00			20.00 ± 1.00	
	Unilateral plank	Upper: 7.00 ± 9.00 Lower: 5.00 ± 10.00		14.00 ± 9.00			16.00 ± 1.00	
Silva et al. (2017) [53]	Partial back squat						~46	No differences were found in ES activation between the exercises.
	Back squat						~44	
Lee et al. (2017) [54]	Front plank	34.93 ± 29.44		34.80 ± 17.51			24.81 ± 7.48	Modified front plank exercises elicited lower EMG activity than traditional planks.
	Unstable front plank	49.82 ± 21.79		51.16 ± 22.98			23.79 ± 5.59	
	Front plank with knees on the floor	15.44 ± 7.94		20.77 ± 8.90			23.54 ± 5.74	
	Front plank with knees on a balance cushion	15.10 ± 8.30		23.54 ± 5.79			23.47 ± 5.35	
Krommes et al. (2017) [55]	Isometric ankle adduction against a ball	~8		~19				These exercises may be considered as core strengthening exercises given the EMG activity reached by each muscle.
	Isometric knee adduction against a ball	~4		~14				
	Folding knife	~83		~100				
	Standing one-leg cross-country skiing	~4		~14				
	Hip adduction against a partner’s hip abduction	~12		~31				
	Hip abduction against a partner’s hip adduction	~10		~25				
Van Oosterwijck et al. (2017) [56]	Prone trunk extension without active lumbopelvic control					57.20 ± 20.40		There were no significant differences in EMG activity based on lumbopelvic control.
	Prone trunk extension with active lumbopelvic control					63.50 ± 21.00		
	Prone leg extension without active lumbopelvic control					64.20 ± 27.50		
	Prone leg extension with active lumbopelvic control					57.10 ± 18.10		
Kim et al. (2016) [57]	Front plank	41.16 ± 18.19	43.52 ± 13.31	34.18 ± 13.17				Front plank with unilateral hip adduction resisted by elastic bands elicited the greatest EMG activation in RA, IO, and EO. The addition of elastic bands to the front plank on the ground increased EMG activity of all the muscles tested compared to the front plank on the ground.
	Front plank with bilateral hip adduction resisted by elastic bands	48.77 ± 18.16	48.68 ± 15.14	40.18 ± 17.80				
	Front plank with unilateral hip adduction resisted by elastic bands	55.46 ± 17.51	55.50 ± 13.14	43.56 ± 17.76				
Kim & Lee (2016) [58]	Sit-up	Upper: 28.50 ± 12.00 Lower: 27.90 ± 9.80		23.10 ± 9.50				The greatest RA and EO activation were observed during sit-up exercises. Specifically, eccentric sit-up increased EMG activity of RA (even greater in upper RA) and EO.
	Eccentric sit-up	Upper: 31.60 ± 13.60 Lower: 34.10 ± 10.40		27.60 ± 11.00				
	Leg raise	Upper: 20.70 ± 13.40 Lower: 21.70 ± 10.60		14.50 ± 9.00				
	Eccentric leg raise	Upper: 23.10 ± 15.20 Lower: 24.70 ± 14.70		15.70 ± 10.70				
Youdas et al. (2016) [59]	Inverted row with pronated grip both feet weight-bearing	23.30 ± 24.70				46.90 ± 21.50		MUL activation was greater than RA. Supinated exercises elicited greater activation than pronated ones.
	Inverted row with supinated grip single-leg weight-bearing	33.10 ± 21.30				41.40 ± 23.30		
	Inverted row with pronated grip one leg weight-bearing	23.60 ± 17.50				46.30 ± 25.30		
	Inverted row with supinated grip one leg weight-bearing	32.70 ± 19.20				43.20 ± 26.20		
Escamilla et al. (2016) [60]	Front plank	Upper: 34.00 ± 15.00 Lower: 40.00 ± 10.00	29.00 ± 12.00	40.00 ± 21.00				The greatest RA EMG activity was observed in the crunch exercise. The addition of the Swiss ball to plank exercises increased EMG activity in all muscles. However, the greatest IO and EO were observed in lateral planks. EMG activity decreased when performing exercises with knees on the ground.
	Front plank on knees	Upper: 27.00 ± 9.00 Lower: 26.00 ± 9.00	20.00 ± 8.00	22.00 ± 14.00				
	Front plank on a Swiss ball	Upper: 49.00 ± 26.00 Lower: 48.00 ± 9.00	39.00 ± 19.00	42.00 ± 23.00				
	Front plank on Swiss ball with hip extension	Upper: 43.00 ± 21.00 Lower: 44.00 ± 11.00	45.00 ± 25.00	56.00 ± 32.00				
	Lateral plank	Upper: 26.00 ± 15.00 Lower: 21.00 ± 9.00	28.00 ± 12.00	62.00 ± 37.00				
	Lateral plank on knees	Upper: 17.00 ± 10.00 Lower: 14.00 ± 8.00	17.00 ± 7.00	37.00 ± 27.00				
	Crunch	Upper: 53.00 ± 19.00 Lower: 39.00 ± 16.00	33.00 ± 13.00	28.00 ± 17.00				
	Side crunch on a Swiss ball	Upper: 21.00 ± 11.00 Lower: 16.00 ± 7.00	20.00 ± 1.00	50.00 ± 26.00				
	Sit-up	Upper: 40.00 ± 13.00 Lower: 35.00 ± 14.00	31.00 ± 11.00	36.00 ± 14.00				
Mello et al. (2016) [61]	Supine bridge on a Swiss ball					~30	~30	EMG activity of MUL and ES is very similar during the supine bridge on a Swiss ball.
De Blaiser et al. (2016) [62]	Front plank	~58	~62	~64		~14		EO, IO, and RA elicited the greatest muscle activation.
Andersen et al. (2016) [63]	Contralateral 1-armed 16 kg-kettlebell swing	18.50 ± 11.40		18.90 ± 18.90			Upper: 41.80 ± 19.50 Lower: 40.90 ± 16.50	Kettlebell swing with one arm resulted in higher EMG activity for the contralateral side of the ipsilateral side of the RA and upper ES in addition to lower EMG activity of the opposite side of respective muscles.
	Contralateral 2-armed 16 kg-kettlebell swing	25.90 ± 21.30		19.30 ± 20.00			Upper: 32.20 ± 16.20 Lower: 36.00 ± 18.00	
	Ipsilateral 1-armed 16 kg-kettlebell swing	44.10 ± 34.80		24.60 ± 28.80			Upper: 29.70 ± 14.80 Lower: 41.40 ± 19.70	
	Ipsilateral 2-armed 16 kg-kettlebell swing	45.30 ± 47.40		18.70 ± 15.10			Upper: 35.60 ± 13.70 Lower: 36.80 ± 14.50	
Youdas et al. (2015) [64]	Bridge					29.20 ± 14.60		Single-leg bridge on BOSU showed the greatest MUL activation. However, all the exercises similarly activated MUL muscle despite the use of stable and unstable surfaces.
	Bridge on BOSU					30.60 ± 15.60		
	Single-leg bridge					32.10 ± 16.00		
	Single-leg bridge on a BOSU					35.90 ± 18.00		
	Bridge with hamstring curl					33.10 ± 17.00		
	Bridge with hamstring curl on a Swiss ball					34.00 ± 18.20		
Calatayud et al. (2015) [65]	Clean and jerk with 20 kg-barbell			26.00 ± 4.70			74.00 ± 4.00	Clean and jerk with water bag exercise elicited the greatest EO and ES activity, particularly in EO.
	Clean and jerk with 20 kg-sandbag			27.00 ± 4.90			70.00 ± 4.20	
	Clean and jerk with 20 kg water bag			60.00 ± 7.90			85.00 ± 4.90	
Mok et al. (2015) [66]	Front plank with hip abduction on suspension device	~40		~70		~38		Front plank with hip abduction on suspension device elicited the greatest EMG activity or RA and EO muscles. However, the greatest activity of MUL was found in the chest press. These results indicate that lower-limb core exercises may elicit greater EMG activity than upper limb exercises.
	Chest press on suspension device	~10		~15		~55		
	Row at 45° on suspension device	~8		~10		~15		
	Hamstring curl on suspension device	~35		~30		~8		
Ha et al. (2015) [67]	Straight one-leg hold (45°)	7.80 ± 3.80	19.90 ± 13.10	19.90 ± 13.10				Straight one-leg hold exercises on a foam roll and motorized rotating platform are more effective to increasing EMG activity of RA, IO, and EO compared to the floor condition.
	Straight one-leg hold (45°) on a foam-roll	13.90 ± 13.10	32.80 ± 22.20	32.80 ± 22.20				
	Straight one-leg hold (45°) on a motorized rotating platform	13.50 ± 9.60	39.70 ± 31.10	39.70 ± 31.10				
Park et al. (2015) [68]	Inverted row						54.91 ± 15.05	Back extension elicited the greatest ES activation.
	Triceps dips						38.23 ± 15.35	
	Two-legged back extension						63.06 ± 16.16	
	Side crunch						52.85 ± 13.82	
Yavuz et al. (2015) [69]	Back squat						43.20 ± 15.60	Front squat elicited greater ES activation than the back squat.
	Front squat						46.20 ± 12.10	
Borreani et al. (2015) [70]	Push-up					3.97 ± 0.43		Greater activation was found in the suspended push-up than in the ground push-up exercise in MUL. The addition of unstable surfaces increased EMG activity, being the most effective suspension system.
	Push-up on a wobble board					5.03 ± 0.59		
	Push-up on a stability disc					4.70 ± 0.52		
	Push-up on a fitness dome					4.40 ± 0.51		
	Suspended push-up					7.35 ± 0.66		
Masaki et al. (2015) [71]	Bird dog					28.50 ± 10.00	22.50 ± 6.60	Bird dog with the load on hand and leg elicited the greatest MUL and ES activity compared to the rest of the exercises. Hip and shoulder abductions resulted in greater MUL activity.
	Bird dog with shoulder abduction					28.20 ± 9.30	19.40 ± 6.30	
	Bird dog with hip abduction					34.10 ± 8.40	19.40 ± 5.70	
	Bird dog with hip and shoulder abduction					33.10 ± 8.0	15.40 ± 4.70	
	Bird dog with the load on hand					32.90 ± 10.20	28.60 ± 8.70	
	Bird dog with the load on leg					33.80 ± 13.10	26.80 ± 8.50	
	Bird dog with the load on hand and leg					38.60 ± 14.50	31.50 ± 9.70	
Moon et al. (2015) [72]	Abdominal drawing-in maneuver	4.62 ± 3.17		14.04 ± 9.90				Roll-up exercise elicited greater RA and EO muscle activation than the rest of the exercises.
	Bridge	24.35 ± 7.68		20.87 ± 9.28				
	Roll-up	27.84 ± 16.50		28.90 ± 14.87				
	One-leg raise	9.10 ± 4.92		16.92 ± 8.29				
Calatayud et al. (2014) [17]	Push-up	23.85 ± 2.80					2.03 ± 0.14	Push-up exercise on suspension systems increased RA and ES activity. In addition, the greatest EMG activity was achieved on an AirFit Trainer Pro, which is a pulley-based suspension system.
	Suspended push-up on TRX	87.98 ± 8.98					3.21 ± 0.24	
	Suspended push-up on Jungle Gym XT	87.13 ± 9.27					3.26 ± 0.23	
	Suspended push-up on Flying	91.11 ± 10.54					3.31 ± 0.24	
	Suspended push-up on AirFit Trainer Pro	105.53 ± 9.84					4.32 ± 0.32	
Badiuk et al. (2014) [73]	Hollowing	~15	~33	~8				The Lewit exercise most increased RA, IO, and EO muscle activity compared to hollowing and bracing.
	Bracing	~22	~34	~12				
	Lewit	~26	~55	~14				
Fletcher & Bagley (2014) [74]	Back squat						113.50 ± 37.10	A significant increase in ES activation was found, which is related to a decrease in squat stability.
	Back squat (Smith machine)						95.70 ± 39.10	
	Back squat with a destabilizing bar						134.10 ± 55.40	
Serner et al. (2014) [75]	Isometric adduction with a ball between the knees	8.30 ± 3.00		13.00 ± 3.00				Copenhagen hip adduction and supine hip adduction elicited the greatest RA and EO activation.
	Copenhagen adduction	40.00 ± 3.00		36.00 ± 3.00				
	Hip adduction with an elastic band	9.00 ± 3.00		18.00± 3.00				
	Hip adduction on an adductor machine	11.00 ± 3.00		15.00 ± 3.00				
	Sliding hip abduction/adduction exercise	7.00 ± 3.00		14.00 ± 3.00				
	Isometric adduction with a ball between the ankles	21.00 ± 3.00		18.00 ± 3.00				
	Side-lying hip adduction	13.00 ± 3.00		21.00 ± 3.00				
	Supine hip adduction	36.00 ± 3.00		35.00 ± 3.00				
Byrne et al. (2014) [78]	Front plank	~22		~15				RA and EO activity levels increased on suspended front planks.
	Front plank with suspended feet	~37		~18				
	Front plank with suspended arms	~60		~30				
	Front plank with suspended feet and arms	~62		~29				
Calatayud et al. (2014) [79]	Push-up	13.31 ± 1.63		25.14 ± 3.80				Suspended push-ups elicited the greatest activity of RA and EO.
	Elastic-resisted push-up	15.48 ± 1.72		30.62 ± 4.06				
	Suspended push-up with closed eyes	57.08 ± 8.38		56.02 ± 6.55				
	Suspended push-up with open eyes	60.04 ± 10.20		55.28 ± 7.43				
	Suspended push-up with a pulley system	65.82 ± 10.12		74.61 ± 6.67				
	Bench press 50% 1RM	1.84 ± 0.30		4.16 ± 0.73				
	Bench press 70% 1RM	3.33 ± 0.53		5.18 ± 0.62				
	Bench press 85% 1RM	4.65 ± 0.62		5.80 ± 0.66				
	Standing cable press 50% 1RM	2.00 ± 0.28		4.55 ± 1.20				
	Standing cable press 70% 1RM	2.17 ± 0.21		5.67 ± 1.22				
	Standing cable press 85% 1RM	3.34 ± 0.44		6.05 ± 1.04				
Calatayud et al. (2014) Spain [80]	Push-up with hands at 10 cm from the floor	~23.84					~2.03	Push-ups at 65 cm from the floor decreased the intensity and muscle activity compared to the 10 cm position.
	Push-up with hands at 65 cm from the floor	~9.36					1.37	
	Suspended push-up with hands at 10 cm from the floor	~105.53					~4.32	
	Suspended push-up with hands at 65 cm from the floor	~87.54					~3.23	
Ishida & Watanabe (2014) [27]	Side plank	~18	~18	~25				A significant increase of RA, EO, and IO activation was observed when performing maximum expirations, specifically, during side planks.
	Maximum expiration	~10	~60	~45				
	Side plank with a maximum expiration	~75	~55	~75				
Andersen et al. (2014) [30]	Back squat	~210		~100			~85	Higher EMG activity in EO was observed when performing Bulgarian squats compared to regular squats. Only the Bulgarian squat reported differences in RA, EO, and ES in relation to the type of surface (stable or unstable).
	Bulgarian squat	~180		~155			~80	
	Unstable back squat	~160		~98			~80	
	Unstable Bulgarian squat	~210		~148			~75	
Czaprowski et al. (2014) [81]	Front plank	18.10 ± 9.10		42.30 ± 19.50				The greatest RA and EO activity was found in the front plank on the Swiss ball exercise. Greater EMG activity was observed as instability increased.
	Front plank on a BOSU	20.40 ± 9.50		44.80 ± 21.30				
	Front plank on a Swiss ball	44.70 ± 19.20		54.70 ± 22.90				
	Side plank	16.10 ± 6.70		37.60 ± 16.30				
	Side plank on a BOSU	18.60 ± 6.80		45.10 ± 20.80				
	Supine bridge	2.16 ± 1.60		3.90 ± 2.30				
	Supine bridge on a BOSU	2.06 ± 1.20		3.80 ± 2.40				
	Supine bridge on a Swiss ball	3.53 ± 2.60		6.60 ± 4.00				
Kim et al. (2013) [82]	Bridge	~21	~20			~39	~46	IO and MUL activity increased when adding instability through the Swiss ball. In addition, IO increased with arm movement to the bridge exercises.
	Bridge with arms motion	~15	~21			~37	~37	
	Bridge on a Swiss ball	~20	~26			~45	~46	
	Bridge on a Swiss ball with arms motion	~21	~40			~44	~39	
Maeo et al. (2013) [25]	Bracing	~18	~60	~27			~18	V-sits elicited the greatest RA and EO activity, while hollowing elicited the greatest activation on IO and back extension on ES. Abdominal bracing also showed high IO activity compared to other exercises, including trunk flexion or extension movements.
	Hollowing	~5	~64	~20			~15	
	Front plank	~35	~25	~25			~5	
	Side plank	~15	~26	~35			~20	
	Bridge	~10	~12	~10			~35	
	V-sits	~80	~52	~66			~7	
	Crunch	~45	~26	~35			~5	
	Sit-up	~43	~38	~47			~8	
	Back extension on the floor	~3	~3	~3			~63	
	Back extension on a bench	~2	~2	~2			~56	
McGill & Masrshall (2012) [33]	16 kg Kettlebell swing	6.90 ± 6.50	42.40 ± 42.50	16.50 ± 12.90			55.40 ± 10.90	Kettlebell swings with Kime and kettlebell swings to the snatch position elicited greater RA, IO, and EO muscle activation than kettlebell swings. Muscle activation in ES and IO was greater than RA or EO during these exercises.
	16 kg Kettlebell swing with Kime	10.90 ± 7.70	80.80 ± 43.70	33.90 ± 31.90			67.20 ± 24.90	
	16 kg Kettlebell swing to snatch position	11.40 ± 11.30	53.60 ± 41.20	33.80 ± 23.40			68.40 ± 13.90	

EMG: electromyographic activity; % MVIC: percentage of maximum voluntary isometric contraction; RM: maximum repetition; °: degrees; BW: body weight; RA: rectus abdominis; IO: internal oblique; EO: external oblique; TA: transversus abdominis; MUL: lumbar multifidus; ES: erector spinae.

**Table 4:** Descriptive statistics of electromyographic activity (expressed as millivolts, mV) in each study by exercise.

Reference	Core Exercise	RA	IO	EO	TA	MUL	ES	Conclusion
Andersen et al. (2019) [40]	Deadlift						~0.34	Deadlift with four elastic bands elicited greater EMG activity compared to deadlift with two elastic bands.
	Deadlift with 2 elastic bands						~0.34	
	Deadlift with 4 elastic bands						~0.36	
Van den Tillaar & Saeterbakken (2018) [28]	Front plank with 20% of extra body mass	~0.25		~0.20			~0.07	Greater RA activation was found during the front plank exercise, but the squat elicited greater ES and external EO activation.
	6RM squat	~0.17		~0.30			~0.35	
Patterson et al. (2015) [34]	Bench press 70% RM		~0.02	~0.01				No significant differences were found between these conditions.
	Unilateral bench press 70% RM		~0.05	~0.02				
	Bench press on unstable bench 70% RM		~0.02	~0.01				
	Unilateral bench press on unstable bench 70% RM		~0.05	~0.02				
Saeterbakken et al. (2014) [16]	10RM Sit-up	Upper: 0.27 ± 0.11 Lower: 0.54 ± 0.24		0.41 ± 0.17				The greatest EMG activity was observed when adding whole-body instability with the BOSU on the feet and low back. In addition, EMG was greater in lower RA than upper RA during sit-ups.
	10RM Sit-up with the feet on BOSU (lower-body instability)	Upper: 0.26 ± 0.11 Lower: 0.53 ± 0.25		0.38 ± 0.16				
	10RM Sit-up with the low back on BOSU (upper-body instability)	Upper: 0.33 ± 0.14 Lower: 0.65 ± 0.33		0.42 ± 0.17				
	10RM Sit-up with the feet and low back on BOSU (whole-body instability)	Upper: 0.33 ± 0.13 Lower: 0.59 ± 0.28		0.44 ± 0.22				
Van den Tillaar & Saeterbakken (2014) [76]	Bench press	~0.15		~0.10			~0.06	Bench press elicited greater activation of the RA than EO and ES.
Saeterbakken et al. (2014) [77]	Back squat	0.06 ± 0.07		0.06 ± 0.05			0.21 ± 0.09	No significant differences were found between these conditions.
	Back squat with elastic bands	0.06 ± 0.08		0.06 ± 0.08			0.21 ± 0.10	
Wagner et al. (2014) [31]	Crunch	~0.09	~0.08	~0.08				Crunch elicited greater RA activity than laughter yoga, but this exercise showed greater IO activation than crunch.
	Climax laughter	~0.04	~0.11	~0.07				
Saeterbakken et al. (2013) [83]	Back squat	0.03 ± 0.01		0.06 ± 0.02			0.31 ± 0.12	No significant differences were found between the exercises. Back squat shows greater ES activation than RA and EO.
	Back squat on power board	0.04 ± 0.02		0.07 ± 0.03			0.29 ± 0.13	
	Back squat on BOSU	0.03 ± 0.01		0.06 ± 0.03			0.25 ± 0.10	
	Back squat on balance cone	0.03 ± 0.01		0.07 ± 0.02			0.28 ± 0.08	
Saeterbakken et al. (2013) [84]	Bench press	0.03 ± 0.03		0.06 ± 0.02			0.14 ± 0.06	RA activation was greater when performing the bench press on a Swiss ball than on a stable bench. The balance cushion elicited greater ES activity than the rest of the exercises.
	Bench press on a balance cushion	0.03 ± 0.02		0.06 ± 0.02			0.16 ± 0.05	
	Bench press on a Swiss ball	0.05 ± 0.04		0.06 ± 0.02			0.13 ± 0.05	
Pirouzi et al. (2013) [85]	Bird dog		1.63 ± 1.35		2.63 ± 3.11	0.86 ± 1.01		Bird dog elicited the greatest activation in transversus abdominis.
Saeterbakken & Steiro (2012) [86]	Seated dumbbell press (bilateral) 80% RM	0.03 ± 0.02		0.06 ± 0.03			0.06 ± 0.04	Greater EMG activity was found when exercises were performed standing compared to seated and unilaterally compared to bilaterally.
	Seated dumbbell press (unilateral) 80% RM	0.02 ± 0.01		0.30 ± 0.15			0.10 ± 0.07	
	Standing dumbbell press (bilateral) 80% RM	0.09 ± 0.05		0.14 ± 0.08			0.08 ± 0.11	
	Standing dumbbell press (unilateral) 80% RM	0.08 ± 0.04		0.42 ± 0.19			0.09 ± 0.12	
Tarnanen et al. (2012) [87]	Trunk flexion	~0.09		~0.09		~0.01		Upper limb exercises can effectively activate core muscles. Bilateral and unilateral shoulder extension, as well as unilateral shoulder horizontal adduction and abduction, elicited the greatest EMG activity in core muscles.
	Trunk lateral flexion	~0.04		~0.09		~0.03		
	Trunk extension	~0.01		~0.03		~0.08		
	Bilateral shoulder extension	~0.08		~0.05		~0.01		
	Unilateral shoulder adduction	~0.05		~0.06		~0.01		
	Unilateral shoulder abduction	~0.01		~0.04		~0.05		
	Unilateral shoulder flexion	~0.01		~0.05		~0.02		
	Unilateral shoulder extension	~0.02		~0.04		~0.05		

EMG: electromyographic activity; % MVIC: percentage of maximum voluntary isometric contraction; RM: maximum repetition; BW: body weight; RA: rectus abdominis; IO: internal oblique; EO: external oblique; TA: transversus abdominis; MUL: lumbar multifidus; ES: erector spinae.
